# Medical Reform—National Convention

**Published:** 1846-02-01

**Authors:** 


### EDITORIAL, MEDICAL INTELLIGENCE, &c.

*Medical Reform—National Convention.*—We have taken occasion in each of our preceding numbers to offer a few desultory remarks on the subject of medical reform. Since all must agree that the existing state of the profession loudly calls for reform, the subject is not only a legitimate one for the Medical Journalist, but one, the importance of which claims for it especial attention. If, however, any apology were required for so constantly referring to it, we might adduce the fact, that, at the present time, the subject is one of peculiar interest, in consequence of an approaching convention of delegates from the different Schools and Associations throughout the Union, to take into consideration means for improving medical education, and elevating the character of the profession.—As regards the meeting of the Convention, we are glad to see in the January number of the New York Journal of Medicine, an assurance from Dr. Davis, (on whose motion the plan was adopted by the New York State Society) that it is now no longer a matter of question that it will be held, and numerously attended. We shall continue, therefore, to throw out such ideas as occur to us, with a view to excite attention toward the subject, and to suggest trains of reflection in the minds of our readers. Our pages, also, are open to free discussions of the various questions which the subject embraces, by any of our correspondents who choose to engage in it. We have no particular plan to advocate, or objects to secure.—We go for reform; and we care not what particular method be pursued, so that it be judicious, and calculated to produce the desired result. We are willing to leave the ways and means to the deliberative wisdom of a body of men composed of the most sagacious, prudent, and high-minded members of the profession, from the different parts of the Union: Of such men, it is to be hoped, the Convention will be composed.

A want of interest in the subject by the profession generally, an apathy often founded on the too hasty belief that nothing can be done to remedy existing evils, is a serious obstacle in the way of reform. We have more fear from this, than from any other source of failure in the proposed method of concerted action. We fear that the profession will not be so fully represented as it ought to be, or that proper care and discrimination will not, in all cases, be exercised in the selection of delegates. The consequence will be, that some interests being, or appearing to be represented more than others, the measures determined on will labor under the imputation of being partial, or partizan in their character. We wish the appeal could be made to every local medical association throughout our country, to make this the subject of formal, deliberative consideration, and not to fail to unite in an object which embraces the general, permanent welfare of the profession. We look upon the present movement as a highly critical one, believing, as we do, that any system to elevate the character of the whole profession, and to establish uniformity in medical education and requirements, must emanate from a national, medical congress, composed of members from every part of our country, representing fairly and fully all interests; and also, believing that if this attempt prove unsuccessful, a long period must elapse before another effort will be made.

Another source of danger is the diversity of opinions respecting the best means of effecting a reform. There is, unfortunately, a great want of unanimity in this respect in the profession. On all subjects there will be honest differences of sentiment, and, more especially on matters involving untried experience, this will necessarily be the case. We trust that different views may be so reconciled and harmonized, that the profession will cheerfully co-operate in whatever plan is adopted. If the character of the Convention should be such as to commend itself to the respect and confidence of the profession, our duty is plainly to accept its conclusions, and submit them to a candid trial. Above all, let not individuals be ready to attribute to those who advocate measures which they do not approve, selfish or unworthy motives. If such feelings be allowed to have much scope, the prospect of an hearty union and co-operation for a common purpose is at an end.

It is to be confessed that the subject of reform, is, in itself, of a complex and difficult character. It is not easy for one who endeavors to examine it with careful attention, and a mind free from any particular bias, to decide what is the best course to pursue. This consideration, while it should enforce the necessity of deliberation and prudence in forming opinions, should, also, suggest the propriety of entertaining due distrust of their correctness; and, at least, of exercising candor and liberality toward those which conflict with them.

When we commenced penning this article we designed to indulge in some reflections on the particular points which seem to us to claim especial attention in deliberating upon the means of reform. The views which we intended merely as introductory, have, however, extended themselves so far as to occupy all the space we can devote to the subject in the present number.

